# A portable gas sensor based on In_2_O_3_@CuO P–N heterojunction connected via Wi-Fi to a smartphone for real-time carbon monoxide determination

**DOI:** 10.1038/s41598-024-64534-2

**Published:** 2024-06-12

**Authors:** Sina Khalili, Mohsen Majidi, Morteza Bahrami, Majid Roshanaei, Tayyebeh Madrakian, Abbas Afkhami

**Affiliations:** 1 Research Center for Health Management in Mass Gathering, Red Crescent Society of the Islamic Republic of Iran, Tehran, Iran; 2 Faculty of Chemistry and Petroleum Sciences, Bu-Ali Sina University, Hamedan, 6517838695 Iran; 3 Department of Computer Engineering, Faculty of Engineering, Bu-Ali Sina University, Hamedan, 6517838695 Iran; 4 Biomedical Engineering Department, School of Electrical Engineering, Iran University of Science and Technology, Tehran, 1684613114 Iran; 5 D-8 International University, Hamedan, Iran

**Keywords:** Portable sensor, Gas sensors, Smartphones, Carbon monoxide, P–N heterojunction, Sensors, Electronic materials

## Abstract

This research presents a compact portable electronic gas sensor that can be monitored through a smartphone application. The smart sensor utilizes three state-of-the-art sensors. The sensors integrate an ESP8266 microcontroller within the same device. This facilitates their integration with the electronics and enhances their performance. Herein, primarily focuses on utilizing the sensor to detect carbon monoxide. This article outlines the fabrication process of a gas sensor utilizing a P–N heterojunction, eliminating the need for a binder. The sensor consists of CuO/copper foam nanowires and hierarchical In_2_O_3_. In order to verify the system’s functionality, it underwent testing with various levels of CO concentrations (10–900 ppm), including particular tests designed to examine the device’s performance in different humidity and temperature circumstances. A mobile application for the provision of monitoring services has been developed at last. To process the information obtained from the gas sensor, an algorithm has been constructed, trained, and integrated into a smartphone for this purpose. This research demonstrated that a smartphone-coupled gas sensor is a viable system for real-time monitoring and the detection of CO gas.

## Introduction

Carbon monoxide (CO) is a toxic gas that can be detrimental when inhaled in large amounts, and its release in outdoor settings contributes to air pollution^1,2^. CO is an odorless, flavorless, and poisonous gas to humans. It often arises from incomplete combustion, leading to the production of CO, which poses a significant risk to human health. CO poses a significant danger due to its potential to create intoxication, which is a major contributor to unpredictable illness and death, particularly in cases of inhalation injury resulting from combustion. In this specific situation, prolonged exposure to high levels of carbon monoxide (25–1000 ppm) causes detrimental cardiac events that have the potential to be fatal. Nevertheless, there have been studies suggesting that even short-term exposure to concentrations of approximately 50 ppm can also result in death. Therefore, it is of the utmost importance to identify the substance and technology that are most efficient in detecting this hazardous gas^3–5^.

As a result, numerous materials were investigated by scientists employing a variety of techniques and methods in an effort to detect this gas and commercialize them as CO sensors. Since CO is produced in residential and household settings, it is critical to develop portable devices that are efficient and cost-effective in these environments. At present, an expanding inclination towards the Internet of Things (IoT) has resulted in a heightened emphasis on gas monitoring systems that are small in size and portability^6,7^. Currently, gas sensors based on semiconductors are exceedingly attractive due to their diminutive size and remarkable sensitivity. Wireless sensor networks (WSN) are designed to enhance the collection of environmental data and facilitate its transfer to smartphones. This allows for the distant surveillance of locations that are both intriguing and challenging to reach^8,9^. Currently, the utilization of metal oxide films for gas sensors in portable devices is being improved. Before achieving complete integration of the metal oxide film with a portable gas sensor, there are still specific limitations and drawbacks that must be addressed. One of the most difficult challenges that gas sensor development and their broad availability possess is the requirement for portable sensors with a high selectivity and ultra-low power consumption. The development of energy-efficient heating and sensing materials is crucial for the portable gas sensor’s design. Until now, the microheater has been utilized to reduce power consumption. To enhance the selectivity of portable gas sensors, it is necessary to increase the surface area of the sensing layer. This is because the chemical reactions involved in the sensing process are directly influenced by the amount of surface area that has access to the target gas.

Metal oxide semiconductors (MOSs) have proven effective in portable detecting target gas. MOS sensors possess the advantages of being cost-effective, having high surface area, easy to operate, ultra-low power consumption, and having a quick reaction and recovery time^10,11^. They also exhibit high stability in both physical and chemical aspects, outstanding electrical performance, and a straightforward and portable design. There are primarily two categories of sensors: ‘Blob’ sensors and electrical sensors. “Blob” MOS sensors consist fundamentally of a patch of metal oxide salts that produce carbon dioxide upon reducing the salt in the presence of monoxide. The reduction of the salt results in a color change to black, which serves as an indicator for the observer^12,13^. Electronic carbon monoxide sensors can be broadly classified into two categories: thermistor-type metal-oxide detectors and electrolytic detectors. The former operates by detecting a temperature-dependent resistance change resulting from the reaction between carbon monoxide and the oxide (i.e., when carbon monoxide contacts an electrode of the device). The latter detects the motion of charge carriers in an electrode surface^14^. Resistive gas sensors that utilize MOS such as ZnO, SnO_2_, In_2_O_3_, Co_3_O_4_, WO_3_, MoO_3_, CuO, Fe_2_O_3_, LaFeO_3_, NiO, etc., have gained significant interest among numerous techniques for detecting CO gas^5,15–20^. The heterostructure MOSs plays a crucial role in the portable gas sensor since it determines various elements of the sensor’s performance, including power consumption, sensitivity, and selectivity. Selecting suitable materials for fabricating a heterojunction that has a robust sensitivity and a high interference factor (IF) towards CO. Copper oxide (CuO) is a highly appropriate substance for fabricating heterojunction (P–N) that can be used in the detection of carbon monoxide (CO). Enhancing the precise manipulation of CuO nanostructure will improve its ability to detect CO, hence enhancing its sensing capability. Research has demonstrated that the exposure of CuO to CO leads to reactive adsorption, surface reduction, and the generation of carbonate^21^. To summarize, CuO possesses the capacity to improve CO-sensing capabilities and reduce the impact of water vapor.

Although significant efforts have been devoted to CuO-based sensors for CO detection, there are still a few unresolved issues: (i) Maintaining the ability of the CuO nanostructure to detect CO; (ii) long recovery time. Recently, there has been a significant focus on improving the efficacy of gas sensing by using CuO-based composite sensors. Some examples include binder-free CeO_2_ coated/Pd-decorated Cu_x_O nanowires, CuO/TiO_2_ heterojunction, and In_2_O_3_/CuO Nanospheres^22–24^. In_2_O_3_-based heterojunction materials have gained significant popularity and usage due to their exceptional properties, including a band gap of approximately 3.1 eV at room temperature, excellent optical transparency (> 80%) in the visible region, n-type conductivity, and a high exciton binding energy of about 60 mV^25,26^. Until now, the majority of research on In_2_O_3_ heterojunction materials has mostly examined bulk materials and agglomerated nanoparticles. However, the aggregation and stacking of structure leads to a decrease in the number of active sites and specific surface area, which hinders the materials’ complete utilization for gas sensing. It is essential to have control over the morphology to improve gas sensing efficiency, particularly in selectivity^27^. Recently, notable advancements have been in controlling the shape and structure of gas sensing materials, spanning from zero-dimensional (0D), two-dimensional (2D), and hierarchical three-dimensional (3D)^28^. The fabrication of a hierarchical structure with the desired shape offers both time and cost advantages. Recently, there has been significant interest in using sacrificial templates and organic surfactants to promote the anisotropic growth of hierarchical In_2_O_3_ in solutions. The addition of an organic surfactant altered the morphology of In_2_O_3_ nanocrystals, transforming them from spherical to nanoflowers, microcubes, and nanorods^29,30^. This shift is likely due to the arrangement of the surfactant molecules in micelles. Surfactants and different concentrations of surfactants were used to produce well-crystalline In_2_O_3_. The formation and growth of In (OH)_3_ crystals were primarily influenced by the attachment of surfactant molecules to specific binding sites on the surface of the crystal. This attachment promoted the aggregation of crystals in the corners of the primary crystallites, resulting in the production of In (OH)_3_ crystals with different shapes. Materials that have hierarchical structures and are not agglomerated always have numerous pore structures and a very high specific surface area, which allows for efficient gas diffusion and reaction.

This study introduces a portable gas sensor device that incorporates heterojunction MOS sensors with processing microcontroller capabilities. Alternatively, we developed a novel hierarchical In_2_O_3_ decorated on CuO nanowires using a hydrothermal process with additive organic surfactant. The heterojunction sensor employed for this study consisted of CuO nanowires on Cu foam as the bottom P-type layer, and hierarchical In_2_O_3_ with exposed facets as the top n-type layer. No binder was used in the fabrication process. This composite has a higher number of active sites and is specifically tailored for the detection of CO gas. The device is compact to ensure convenient portability. This gadget provides wireless connectivity with a smart device, specifically a smartphone, and is controlled using an Android application. The investigations were conducted utilizing varying concentrations of CO, which is a highly toxic pollutant present in the indoor atmosphere.

## Results and discussion

### Characterization

**Figure 1:**
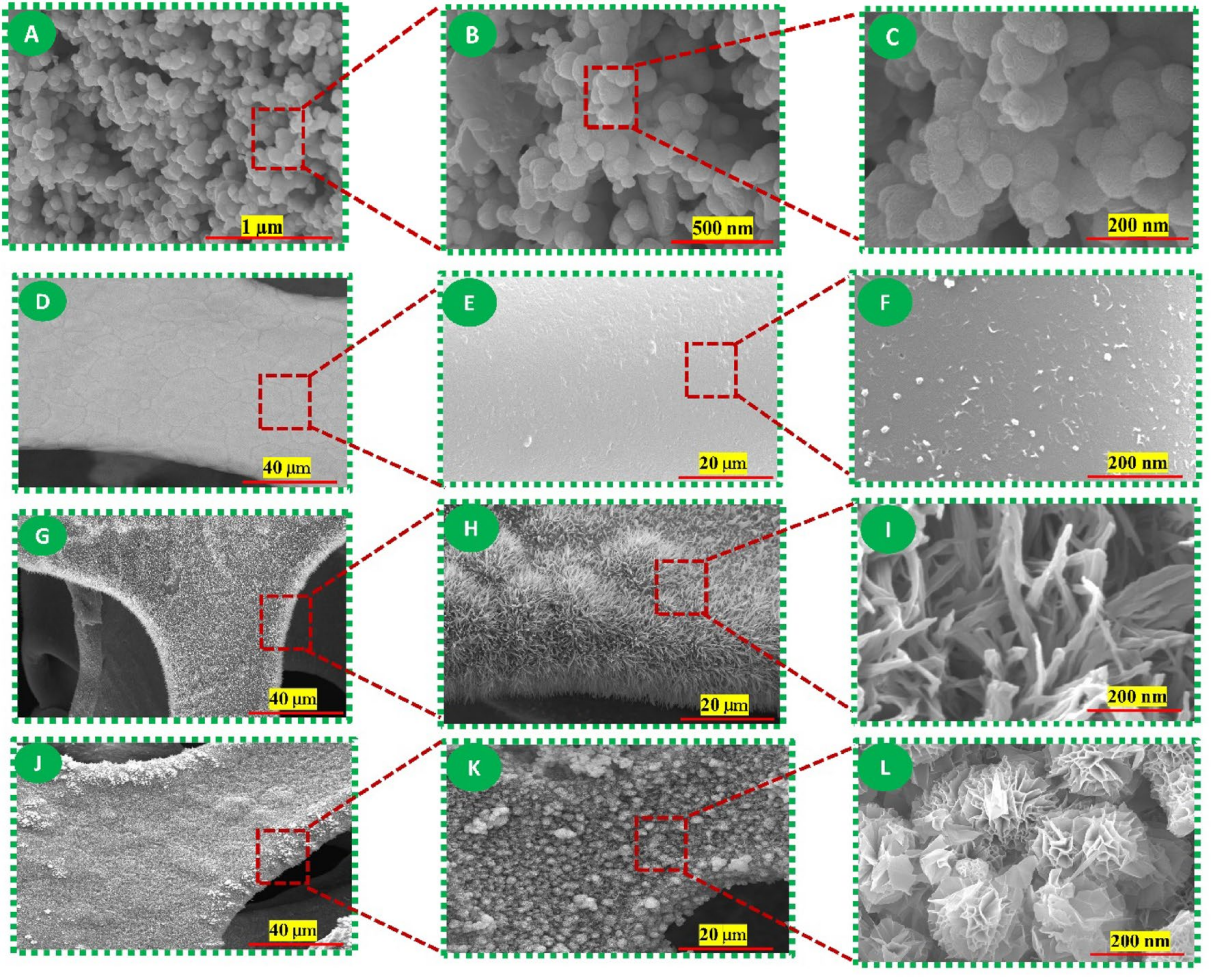
The FE-SEM images exhibiting the morphology of the fabricated materials. (**A**–**C**) pure In_2_O_3_ nanospheres. (**D**–**F**) commercially CF. (**G**–**I**) CuO/CF. (**J**–**L**) In_2_O_3_@CuO/CF.

The morphology of the gas sensor materials has been analyzed using field emission scanning electron microscopy (FE-SEM). Figure 1A–C, displays a representative FESEM image of the pure In_2_O_3_. The produced pure In_2_O_3_ materials exhibit distinct nanospheres in their morphology. Also, the FESEM images are utilized to determine the dimensions of the nanospheres. Figure 1D–F, displays the FE-SEM image of a commercially available copper foam (CF). Various FE-SEM images of CF at varying magnification levels reveal a uniformly even surface appearance. The suggested approach yields pure CuO/CF with a distinct nanorod structure consisting of tightly packed wires (Fig. 1G–I). The magnification of the CuO FE-SEM image clearly illustrates the transformation of CF into bunch-like CuO nanowires after the calcination procedure. The uppermost materials layer consists of a n-type hierarchical In_2_O_3_. Following the hierarchical In_2_O_3_ procedure in the InCl_3_ solution, the resulting In_2_O_3_@CuO/CF material displays a uniform structure consisting of interconnected nanosheets and an empty interior (flower-like structure), as depicted in Fig. 1J–L. The presence of a flower-like structure can accelerate the diffusion of CO gas, leading to improved performance. The FE-SEM image reveals the prominent surface area and easily accessible pores of In_2_O_3_@CuO/CF.

**Figure 2:**
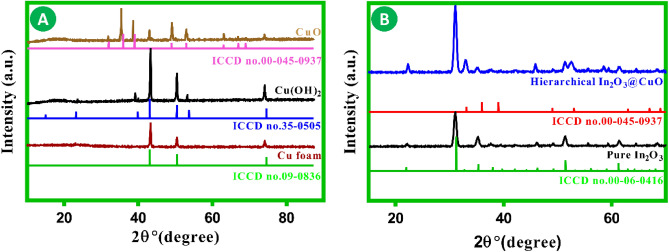
XRD pattern of gas sensor materials.

X-ray diffraction (XRD) research was conducted to provide additional confirmation of the successful assembly of the In_2_O_3_@CuO/CF. The XRD spectrum analysis of the materials is compared during the synthesis processes, as depicted in Fig. 2A,B. The commercial CF exhibits three prominent peaks at 2θ positions of 43.4°, 49.9°, and 73.9°, which can be attributed to the (1 1 1), (2 0 0), and (2 2 0) crystallographic planes, respectively. These peaks are in good agreement with the metallic Cu (ICCD No. 09-0836) present in the CF substrate^31,32^. The XRD patterns of Cu (OH)_2_ exhibit distinct diffraction peaks at 15.9°, 23.8°, 39.5°, 54.7°, and 56.2°, which can be attributed to the (0 2 0), (021), (0 3 0), (2 0 0), and (1 5 0) crystallographic planes, respectively. These peaks are in accordance with the Cu (OH)_2_ (ICCD No. 35-0505) reference pattern, demonstrating the successful synthesis of Cu (OH)_2_^33^. After undergoing calcination, the XRD pattern exhibited notable alterations, including the emergence of clearly identifiable new diffraction peaks at angles of 33.7°, 35.5°, 37.7°, 63.3°, 64.7°, and 69.3°. The observed peaks can be attributed to the (1 1 0), (0 0 2), (1 1 1), (2 0 2), (1 1 3), and (3 1 1) crystallographic planes of the synthesized CuO (ICCD No. 00-045-0937)^34,35^. As can be seen in Fig. 2B, the diffraction pattern was analyzed and matched with the ICDD file No. 06-0416, which corresponds to the pure In_2_O_3_ nanospheres. The determined average size of the crystallites is 19 nm. This indicates that the synthesis process used may readily produce pure In_2_O_3_ nanospheres. The main diffraction peaks at angles of 22.1°, 31.0°, 36.1°, 51.9°, and 61.2°, which can correspond to the (2 1 1), (2 2 2), (4 0 0), (4 4 0), and (6 2 2) crystallographic planes, respectively^36^. The main diffraction peak of In_2_O_3_@CuO/CF can be attributed to the body-centered cubic phase of In_2_O_3_ (ICDD file No. 06-0416). Aside from the peaks resulting from In_2_O_3_, there are a few weak diffraction peaks that can be ascribed to CuO (ICCD No. 00-045-0937). Indications point to the coexistence of CuO and In_2_O_3_. Furthermore, the low intensity of the diffraction peaks indicates that copper is situated as the underlying layer, while hierarchical In_2_O_3_ is positioned as the uppermost layer. This assertion can be corroborated by examining the gas sensor n-type response. In the final sensing materials layers, there has been a minimal presence of impurities. Son et al. have previously reported on the negative effect of impurities on the performance of sensing materials^37^. Synthesis methods are equally significant as concentration reagent and organic surfactant, temperature, and solvent media in determining the final crystallographic planes of the P–N heterojunction. Therefore, careful selection of synthesis method is crucial. Organic materials (such as surfactant and so on) is considered undesired materials in the sensing layer, as it physically blocks the active sites when it deposits on the surface. Employing annealing steps in synthesis methods significantly decreases the level of this impurity in the final crystallographic planes.

### Gas sensing performance

The key performance attributes of a gas sensor encompass its capacity to selectively detect certain gases, sensitivity to low gas concentrations, sensor response, operating temperature, response and recovery times, stability, reversibility, humidity dependence, and fabrication cost^11,38,39^. The operational temperature of a gas sensor refers to the temperature at which its sensitivity reaches its highest level. The signal of an MOS in response to a given gas analyte often shows a bell-shaped curve with a peak at a specific temperature, which is influenced by temperature. The electrical charge of oxygen species that are attached to the surface of the oxide modulates with changes in temperature. The oxidation reaction is a process that is activated by temperatures and its rate is directly related to the temperature. The temperature ultimately determines the adsorption, desorption, and diffusion processes^11,40^.

**Figure 3:**
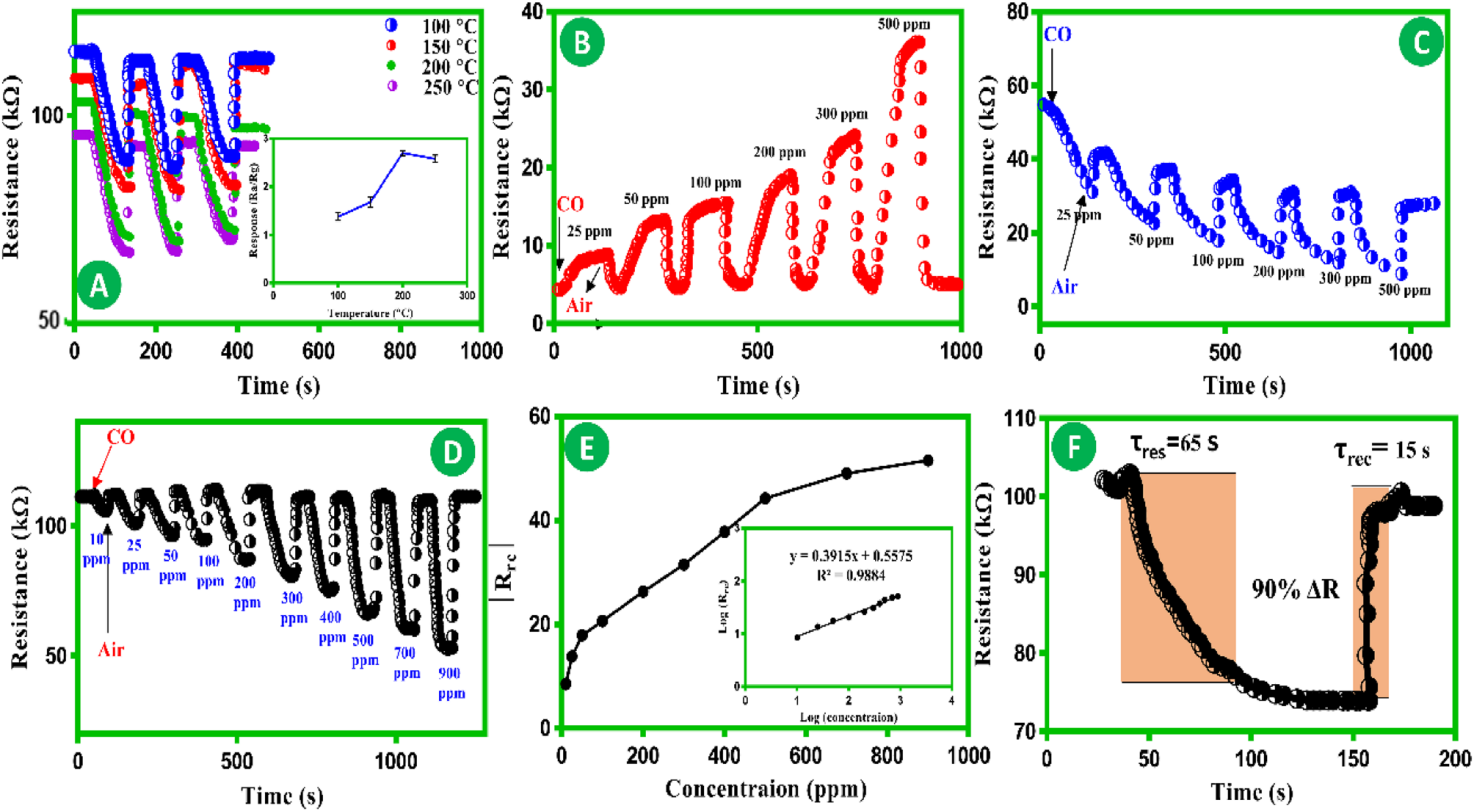
(**A**) Dependence of responses on different operating temperatures at a relative humidity of 30% at 25 °C (300 ppm CO). Resistance and response curves at an operating temperature of 200 °C and a relative humidity of 30% at 25 °C for (**B**) CuO/CF. (**C**) Pure In_2_O_3_ nanospheres. (**D**) hierarchical In_2_O_3_@CuO/CF. (**E**) Relationship between responses and CO concentrations. (**F**) Response/recovery times toward 300 ppm CO at an operating temperature of 200 °C and a relative humidity of 30% at 25 °C.

As can be seen in Fig. 3A, the response values of In_2_O_3_@CuO/CF to a concentration of 300 ppm of CO (3 cycles) according to the operating temperature. The sensor response demonstrates an increase and maximum at a temperature of 200 °C. Subsequently, there is a decline accompanied by a subsequent rise in the operating temperature. Hence, it was concluded that a temperature of 200 °C is the most favorable operating condition for In_2_O_3_@CuO/CF sensors during the subsequent gas-sensing experiments. On the other hand, the sensor’s response is determined by the ratio of the resistance value when exposed to air compared to the resistance value when exposed to CO (Ra/Rg). This curve illustrates the relationship between (Ra/Rg) and temperature throughout the three response cycles. On the other hand, CuO operates as a p-type semiconductor, and its sensing mechanism for reducing gases such as CO involves the modulation of the width of the hole accumulation layer. Upon exposure to air, CuO nanostructures undergo a chemical reaction where oxygen species (O^2–^ and O^–^) attach to the surface of CuO. This process results in the confinement of electrons in the conduction band and the formation of a layer of accumulated holes. Consequently, the resistance of CuO reduces due to the adsorption of oxygen. When the sensing material comes into contact with CO gas, the gas molecules interact with the oxygen species that are chemically absorbed on the surface of nanostructures. This interaction causes the release of electrons back to the CuO, resulting in an increase in resistance. Figure 3B, illustrates the transient response of the CuO/CF nanowires sensor towards CO concentrations ranging from 25 to 500 ppm at a temperature of 200 °C and a relative humidity of 30% at 25 °C. The pure In_2_O_3_ nanospheres sensor exhibits a response of 25 to 500 ppm CO gas at a temperature of 200 °C and a relative humidity of 30% at 25 °C. This level of response is considered increased compared to CuO/CF materials that sense CO gas at the same temperatures (shown in Fig. 3C). Combining the p-type CuO and n-type hierarchical In_2_O_3_ will lead to the formation of an interface between the two layers, which will result in the generation of a space charge region. Consequently, the P–N heterojunction sensor exhibited more significant alterations upon exposure to CO. On the other hand, when CO enters the hierarchical In_2_O_3_@CuO/CF sensor materials, the oxygen ions on the surface are gradually depleted by the catalytic oxidation process for CO. Consequently, the liberation of electrons occurs, leading to their return to the conduction band. This process causes a reduction in the thickness of the depletion layer and a decrease in resistance. This results in the attainment of resistance modulation. The enhanced performance of the In_2_O_3_@CuO/CF in different CO concertations (10–900 ppm) can also be attributed to the increase in oxygen vacancies, in contrast to the monolayer of CuO (shown in Fig. 3D). On the other hand, the gas sensing performance of the In_2_O_3_@CuO/CF is enhanced by the active sites given by its nanomorphology. The resistance in P–N heterojunction consists of the resistance of the p-type (CuO), the junction resistance, and the resistance of the n-type layer (hierarchical In_2_O_3_). Therefore, the electrons will transition from the conduction band of hierarchical In_2_O_3_ to establish p–n junctions at the interface of CuO. Figure 3E, illustrates the correlation between the relative resistance change (Rrc) and the concentrations of CO (ranging from 10 to 900 ppm) for the In_2_O_3_@CuO/CF sensor at a temperature of 200 °C and a relative humidity of 30% at 25 °C.

1 $${\text{Relative}}\,{\text{ resistance }}\,{\text{change }}\,\left( {{\text{Rrc}}} \right) = \left[ {\left( {\frac{{R_{E - } R_{B} }}{{R_{B} }}} \right) \times 100} \right]$$

The Rrc was determined by applying the equation provided in Eq. (1). The symbol Rrc represents the ratio of resistance, where R_B_ is the resistance in an atmosphere containing pure N_2_, and R_E_ is the resistance value when exposed to the CO gas being measured. In this work, a “n-type response” will be used to describe a negative Rrc change in resistance. On the other hand, if the Rrc change in resistance is positive, it will be called a “p-type response”. The │ Rrc│ of the In_2_O_3_@CuO/CF sensor increases proportionally with the concentration of CO (shown in Fig. 3D). Nevertheless, the sensors exhibit a fast increase in their responses when exposed to low levels of CO. Over time, the responses stabilize and eventually reach a condition of near saturation, which can be attributed to the proportion of oxygen ions and CO molecules present. The diagram presented in inset Fig. 3E, shows the logarithm correlation between the Rcr and concentrations of CO (ranging from 10 to 900 ppm). The logarithmic form will exhibit a linear relationship between Rcr and concentrations of CO. The results demonstrate that the correlation coefficient (R^2^) for In_2_O_3_@CuO/CF exceeds 0.988. These findings indicate that the In_2_O_3_@CuO/CF gas sensors exhibit an excellent linear correlation throughout an extensive range of CO gas concentrations.

The response time of a gas sensor refers to the duration it takes for the signal to reach 90% of the total change. The recovery time refers to the duration it takes for the sensor to return to its baseline signal once the target gas has been removed upon its introduction^38^. The real-time response-recovery time at 300 ppm concentrations of CO is demonstrated using gas sensors made of In_2_O_3_@CuO/CF materials (shown in Fig. 3F). The experiments were conducted at optimal temperatures under relative humidity of 30% at 25 °C. According to the observation, the time it takes for In_2_O_3_@CuO/CF to respond and recover from 300 ppm CO is just 65 and 15 s, respectively. It should be noted that the concentration with the longest response and recovery time was selected.

**Figure 4:**
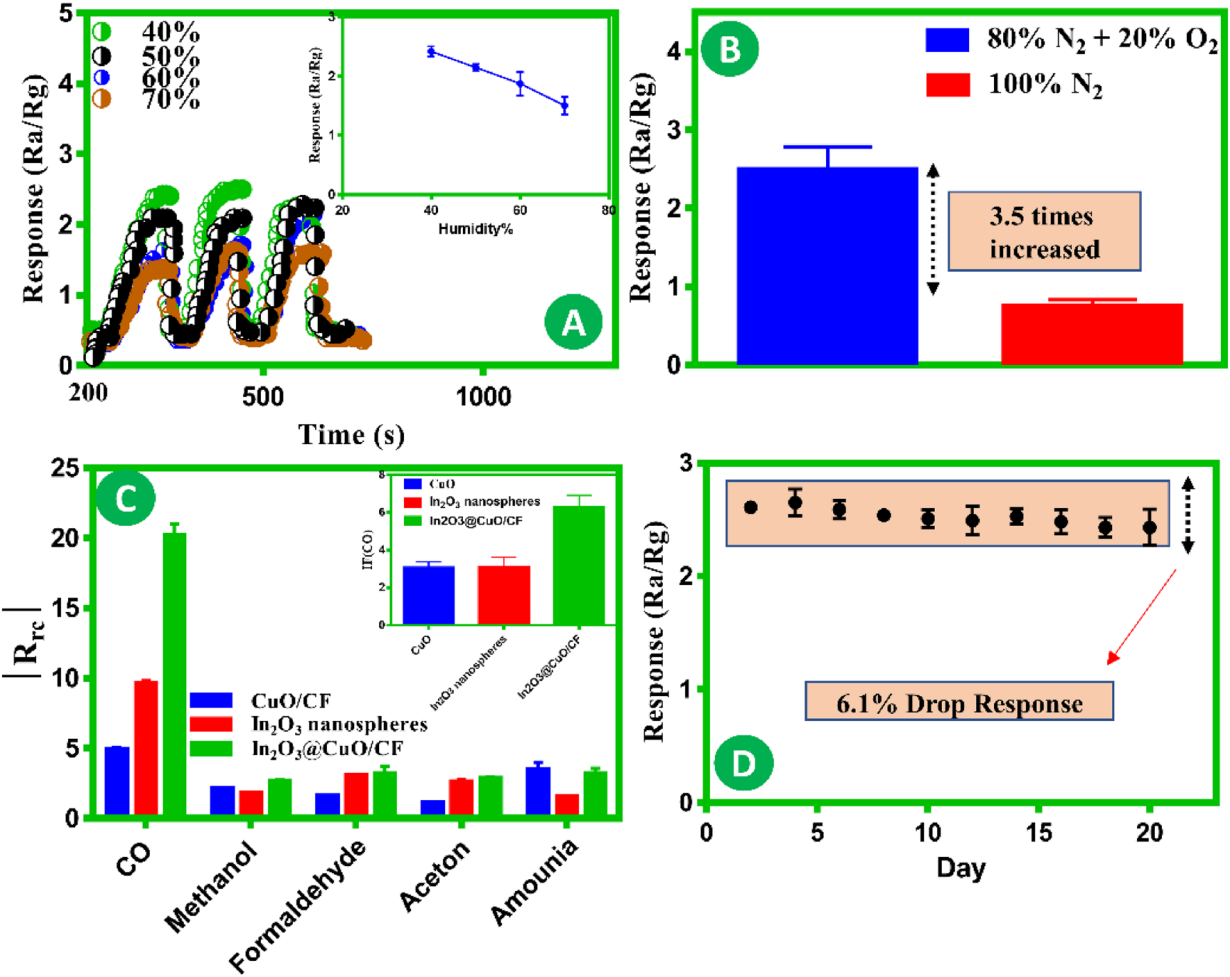
(**A**) Comparative analysis of the response curves of sample hierarchical In_2_O_3_@CuO/CF to 300 ppm CO gas over different humidity situations at an operating temperature of 200 °C. (**B**) Response of hierarchical In_2_O_3_@CuO/CF to 300 ppm CO gas in dry (80% N_2_ + 20% O_2_) and 100% N_2_ atmosphere at an operating temperature of 200 °C and a relative humidity of 30% at 25 °C. (**C**) Response of the CuO/CF, Pure In_2_O_3_ nanospheres, and hierarchical In_2_O_3_@CuO/CF sensors to 100 ppm CO, methanol, formaldehyde, acetone, and ammonia and the IF (CO) of the sensor at an operating temperature of 200 °C and a relative humidity of 30% at 25 °C. (**D**) long-term stability of hierarchical In_2_O_3_@CuO/CF to 300 ppm CO gas at an operating temperature of 200 °C and a relative humidity of 30% at 25 °C.

It is crucial to investigate the ability to detect gas at different humidity levels, as humidity significantly impacts the performance of gas sensors in natural environments^41–43^. Clearly, the humidity level has a significant impact on the response to the gas sensor. Excessive humidity can hinder the functionality of the gas sensor and reduce its service life. Therefore, it is necessary to determine the sensor’s ability to resist moisture^44^. Figure 4A, demonstrates the impact of relative humidity on sensor performance. Increasing the relative humidity from 40% to 70% results in a reduction in the sensor’s response. Nevertheless, as the relative humidity is above 70%, the reaction steadily diminishes. The inhibitory effect on the CO gas sensing processes can be ascribed to the high relative humidity. This experiment aimed to operate at high humidity conditions to observe the genuine impact of humidity. The high relative humidity can cause an inhibiting effect on the processes involved in detecting CO gas. As can be seen in Fig. 4B, the sensor has been evaluated by introducing it to CO gas in an environment of 100% N_2_ gas, and 80% N_2_ and 20% O_2_ for verification. When the sensor detects CO gas in an N_2_ atmosphere, it exhibits a response approximately 3.5 times smaller than in a mixture atmosphere due to the absence of extra CO gas adsorption caused by O_2_ gas adsorption. The selectivity of a gas sensor is a crucial quality from a practical standpoint. To determine the specificity of the gas sensors that were fabricated, their reactions to different gases at a concentration of 100 ppm were measured at a temperature of 200 °C and a relative humidity of 30% at 25 °C (shown in Fig. 4C). The enhanced In_2_O_3_@CuO P–N heterojunction catalytic results in shorter response and recovery times, while also improving the sensor’s selectivity by favoring specific gas reactions over other gases. Utilizing hierarchical as P–N heterojunction effectively reduces sensor cross-sensitivity to interfering gases, a crucial factor for ensuring precise gas detection and quantification. The increased surface area of the material allows for more interactions with the analyte. The hierarchical form of the surface also provides several active sites for the selective binding of the target analyte. Additionally, the binding action may be easily converted into a detectable signal, making it convenient for processing. The hierarchical In_2_O_3_@CuO/CF sensors clearly demonstrate exceptional selectivity towards CO gas. The interference factors (IF) for CO are calculated and shown in Fig. 4C. It is evident that the formation of the heterojunction led to a substantial increase in the value of IF(CO). The Eq. (2), defines the IF (gas_1_, gas_2_) for a specific concentration of CO.

2 $$IF\left( {CO} \right) = \frac{{R_{CO} }}{{R_{Inter} }}$$

R_CO_ and R_Inter_ represent the corresponding responses to CO and interference gas. Here, with the optimal temperature obtained through previous tests, the selectivity in detecting CO gas surpasses that of other gases^45^. On the other hand, this gadget also has an integrated micro-heating element, which is electrically separated from the sensing material, and maintains the operating temperature of the sensor. A method has been developed to improve the selectivity of portable gas sensors by utilizing a measurement technique that involves the thermal modulation of the sensor heater.

Various factors influence the stability of a gas sensor: (A) the stability of the active materials, (B) the surrounding environmental factors such as humidity and temperature, and (C) The properties of the gas being targeted (reducing or oxidizing gas and its concentration). In order to evaluate the stability of a sensor, it is required to test two specific factors: the stability of the conductivity, commonly referred to as the sensor’s baseline; and the stability of the response^46^. Typically, nanostructured oxides with nanograins, as well as nanorods, are prone to deterioration because of their high reactivity. Calcination and annealing can somewhat enhance stability as post-processing treatments, along with lowering the working temperature of the sensor element. Conservative stability refers to the capacity of a sensor to maintain its sensitivity and selectivity over time under standard storage conditions, such as room temperature and ambient humidity. Additionally, the long-term durability of the gas sensor is documented in Fig. 4D.

During the 20-days observation period, both the reference resistance in air and the reaction remain relatively constant, with minor variations over time and a gradual decrease towards the conclusion of the period. The decrease in sensor response can be attributed to the following factors. The In_2_O_3_@CuO/CF can undergo a reaction with some undesirable compounds, resulting in a state of instability. A chemical substance and gas that is not the target gas is commonly referred to as a disturbance, as it induces a reversible reaction. However, it is classified as poisonous if it triggers an irreversible reaction. The three primary poisoning methods are toxicant adsorption, toxicant-induced surface reconstruction, and compound synthesis between the toxicant and catalyst materials. On the other hand, humidity is an essential and unavoidable interfering gas in MOS sensors. Variations in humidity levels in the surrounding environment result in long-term alterations in the baseline resistance. Eq. (3), illustrates a standard chemisorption reaction involving water vapor. In this equation, M_m_ represents the metal sites on the surface, O_ads_ represents the adsorbed oxygen, and S represents the site for chemically adsorbed oxygen.

3 $$H_{2} O + 2M_{m} + O_{ads}^{ - } \leftrightarrow 2\left( {M_{m}^{ + } - OH} \right) + e^{ - } + S$$

The interaction of the target gas with the sensing surface layer depends on different factors including the type of MOS (p-type/n-type), morphology, operating temperature, target gas concentration, and gas diffusion depth. Gas sensing kinetics vary based on analyte molecules and sensing surface parameters. The sensing kinetics, which are influenced by activation energy, transient time constants, gas diffusion, reactivity, reaction rate, surface covering, gas concentration, and operating temperature, determine each gas’s response and recovery behavior. Eley–Rideal mechanisms have been introduced in the past to understand and formulate the gas-sensing phenomenon^46,47^. The kinetics of gas sensing depend extensively on the operating temperature for controlling the response time constant. The functional dependence for a given concentration can be represented as

4 $$\frac{1}{2} O_{2} + S_{ads} + e^{ - } \underset{{k_{d} }}{\overset{{k_{a} }}{\rightleftharpoons}} O_{ads}^{ - }$$

5 $$O_{ads}^{ - } + target \,gas \mathop{\longrightarrow}\limits_{{K_{r} }}^{\phantom{a}}product + S_{ads} + e^{ - }$$

S represents the surface adsorption site, k_a_ and k_d_ are the kinetic rate constants for adsorption and desorption for oxygen molecules, and k_r_ is the kinetic reaction rate constant for target gas molecules.

According to a recent paper^48^, the relationship between the gas concentration and the response time constant for operating temperature can be expressed as

6 $${\uptau }_{res} = { }y{ } \times { }C_{V}^{ - \beta }$$

The equation with constants γ and β can be written as:

7 $${\uptau }_{res} = { } - {\upbeta }\,{ }\ln \,C_{V} + { }\ln \,y$$

**Figure 5:**
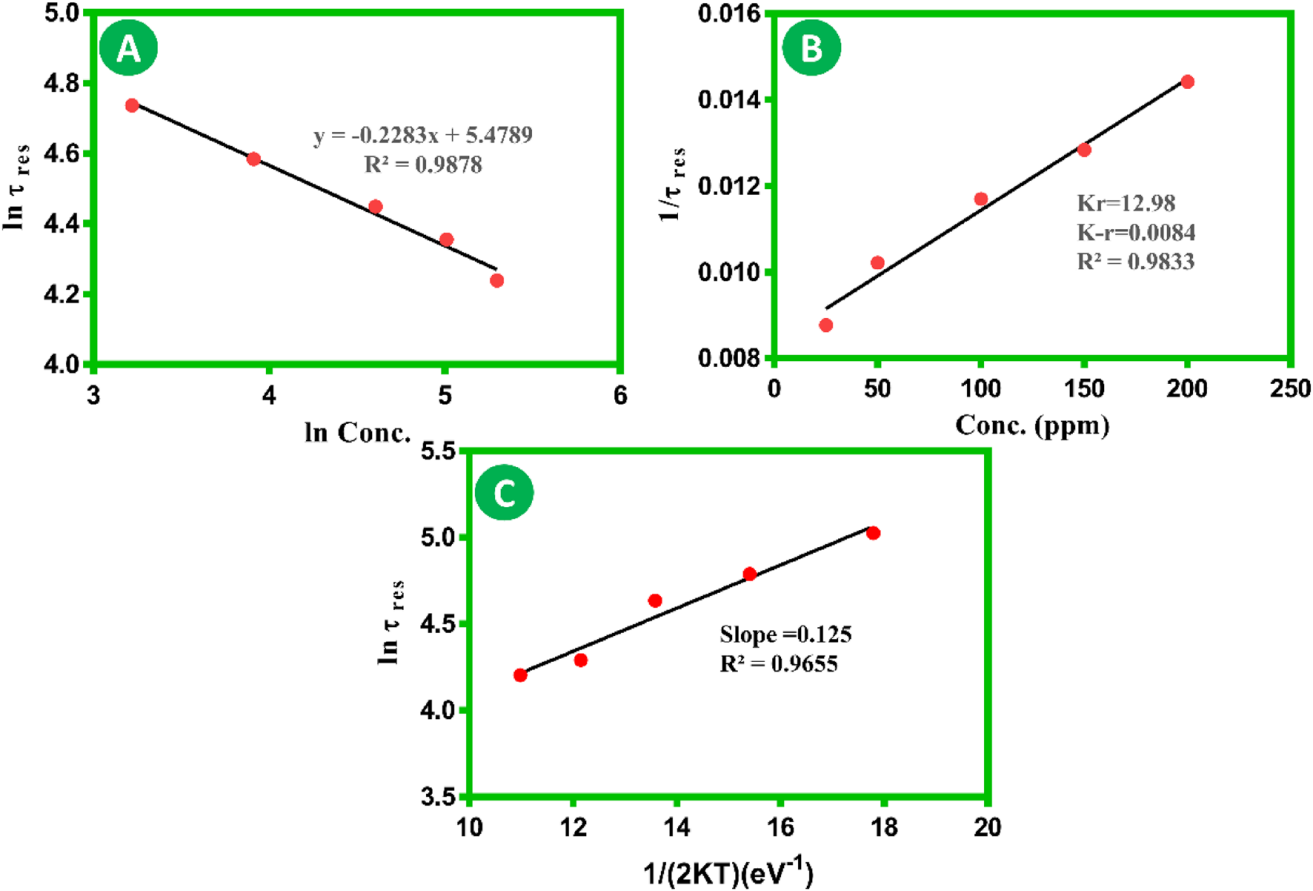
(**A**) ln(τ_res_) vs ln(concentration) plot to obtain β values for tested CO gas at an operating temperature of 200 °C and a relative humidity of 30% at 25 °C. (**B**) 1/τ_res_ versus concentration plot to obtain the kinetic rate constant for tested CO at an operating temperature of 200 °C and relative humidity of 30% at 25 °C. (**C**) Functional dependence of the response time constant over different temperatures to obtain activation energy (Ea) values for tested CO gas at 200 ppm.

Figure 5A, shows a plot of ln (τ_res_) versus ln (C_V_) for optimal temperature. β values, a gas sensing parameter utilized for CO discrimination, were estimated using linear fitting. The negative slopes (β values) obtained were 0.228. The kinetic reaction rate constant (k_r_) for CO gas was also found for the optimal temperature by reverting to Eq. (7). The kinetic rate constant values have been found using the plot between 1/τ_res_ and concentration, as seen in Fig. 5B, where the opposite of the kinetic reaction rate constant (k_−r_) is indicated by the “intercept” with the y-axis, and the kinetic reaction rate constant (k_r_) is represented by the “slope”.

On the other hand, kinetics is greatly influenced by the operating temperature. For a specific concentration, the relationship between variables can vary and be formulated as

8 $${\uptau }_{res} = v^{ - 1} exp\left[ {\frac{{ E_{a} }}{2KT}} \right]$$

$v$, the interacting molecule’s vibrational frequency, Ea, the reaction’s activation energy, K, Boltzmann’s constant, and T, the operating temperature in Kelvin, are all represented in the equation above. CO interaction’s activation energy was calculated for 200 ppm using Eq. (8), as seen in Fig. 5C. This was done by calculating the slope of the plot between the natural logarithm of τ_res_ and 1/(2KT)^−1^. The E_a_ (slope = 0.125) values were calculated from the plots.

As can be seen in Table 1, a comparison of the carbon monoxide (CO) detection abilities of the hierarchical In_2_O_3_@CuO/CF that have been presented with the sensing materials that have been previously documented.

**Table 1:** Comparison of the gas sensing performance of the presented hierarchical In_2_O_3_@CuO/CF with previously reported sensing materials.

Material	Conc. (ppm)	Temp. (°C)	Target gas	Res./Rec. times	Refs.
CuO nanosphere	10–100	150	CO	10 min/15 min	^49^
In_2_O_3_/CuO NW	100–1000	300	CO	10 s/15 s	^50^
Cu_2_O/CuO	10–500	240	CO	4 min/6 min	^51^
AuNP-decorated In_2_O_3_	0.2–5	RT	CO	130 s/50 s	^52^
In/Pd:SnO_2_	10–200	140	CO	15 s/20 s	^53^
SnO_2_/In_2_O_3_	10–100	250	CO	30 s	^54^
In_2_O_3_@CuO/CF	10–900	200	CO	65 s/15 s	This work

**Figure 6:**
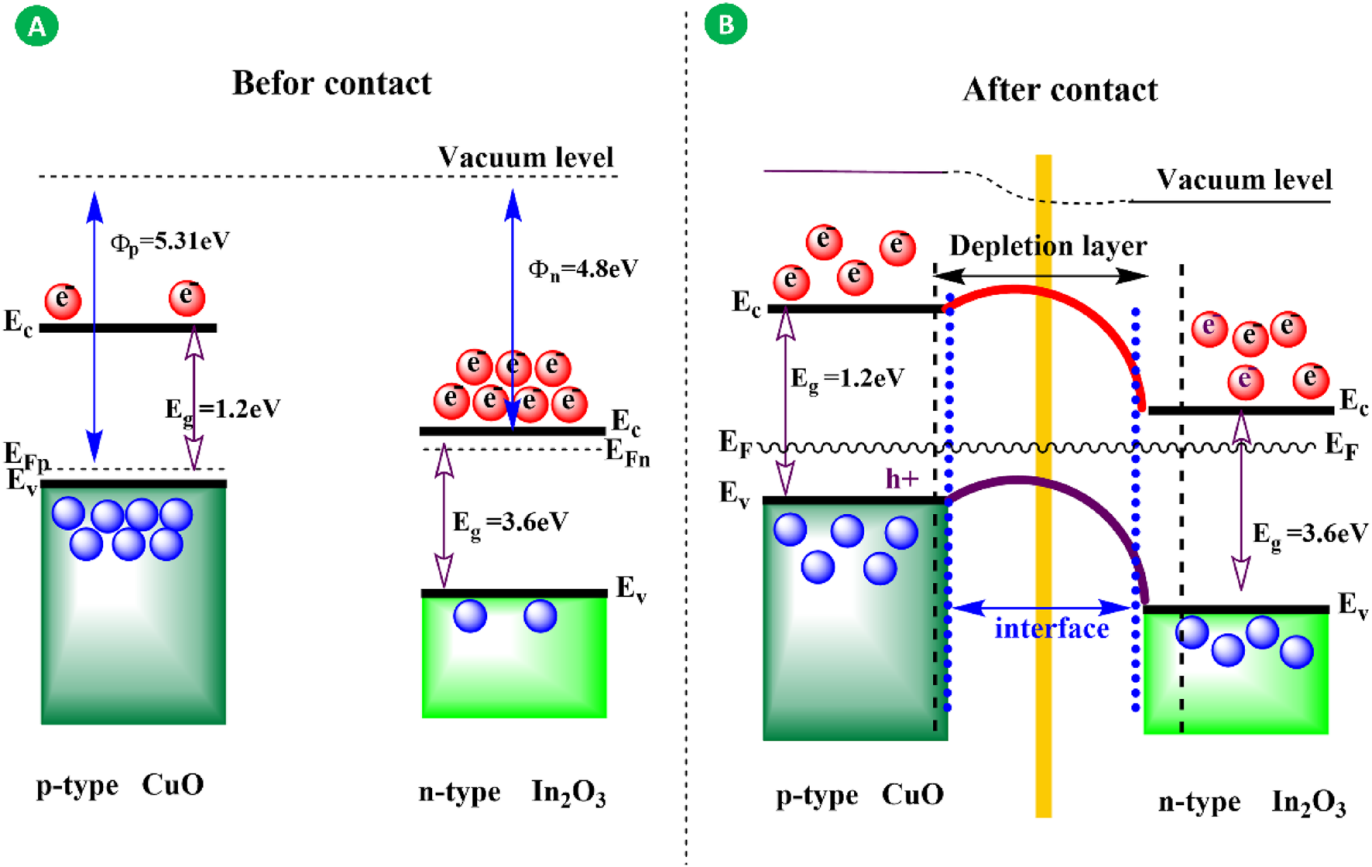
(**A**) Energy band diagram of p-CuO and n-In_2_O_3_. (**B**) Energy band diagram of p-CuO/n-In_2_O_3_ heterostructure with a depletion layer at the interface.

To enhance comprehension, a graphical schematic was employed to examine the mechanism of p–n heterojunction. Figure 6A, demonstrates that CuO is a p-type semiconductor characterized by a small band gap energy of 1.2 eV and a work function of 5.31 eV. On the other hand, In_2_O_3_ has a work function of 4.8 eV and a large band gap energy of 3.6 eV. A p–n heterojunction forms at the interface when these two metal oxides make contact. An electron flow happens in one direction, from In_2_O_3_ to CuO, until both systems reach a state of balance and create a new Fermi energy (Ef) (Fig. 6B). Ultimately, the interface between CuO and In_2_O_3_ results in the formation of a depletion layer. Based on the obtained data, it was determined that the hierarchical In_2_O_3_@CuO/CF exhibits superior performance in detecting CO compared to the pure In_2_O_3_/CF and CuO/CF sensors. The cause can be ascribed to the p–n heterojunction established at the contact between CuO and In_2_O_3_.

### Portable gas sensor device and android application

**Figure 7:**
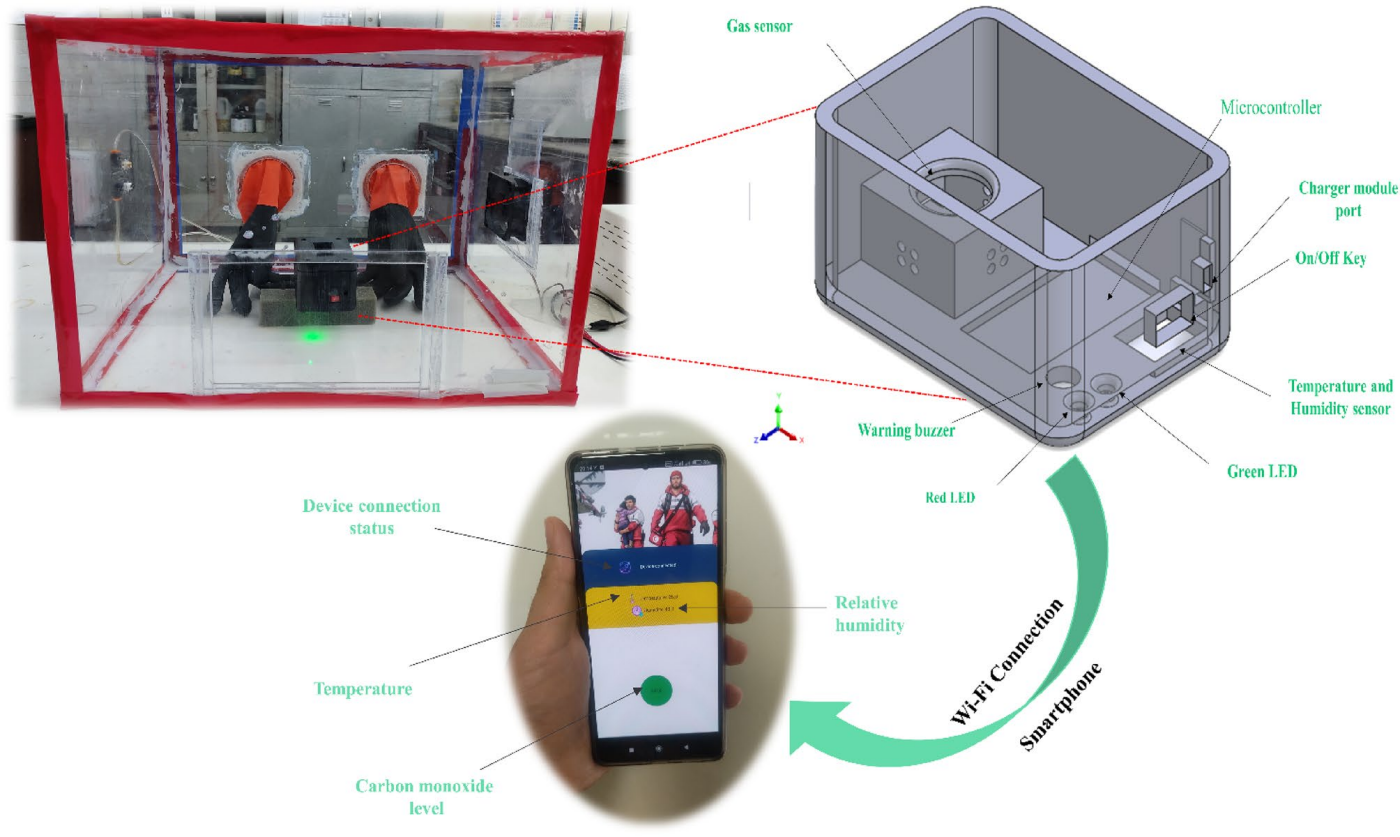
Schematic depicting the arrangement and constituents of the portable gas detection device and Android application.

The design of the device was initially developed utilizing Solidworks software, followed by its fabrication via a 3D printer. The printed design incorporates every component of the portable sensor, as illustrated in Fig. 7. Efficient installation of all components has been achieved, and every effort has been made to prevent any enlargement of the device’s dimensions. Additionally, each component’s location is indicated in Fig. 7. The device consists of a battery, a charging module, a node-MCU ESP8266 microcontroller, a gas sensor, a DHT11 temperature and humidity sensor, two LEDs, and an alarm buzzer. In this smart environmental monitoring system, an ESP8266 microcontroller interfaces with a designed gas sensor and a DHT11 temperature and humidity sensor to ensure real-time assessment of indoor air quality. Under typical conditions, a green LED signals a safe environment.

**Figure 8:**
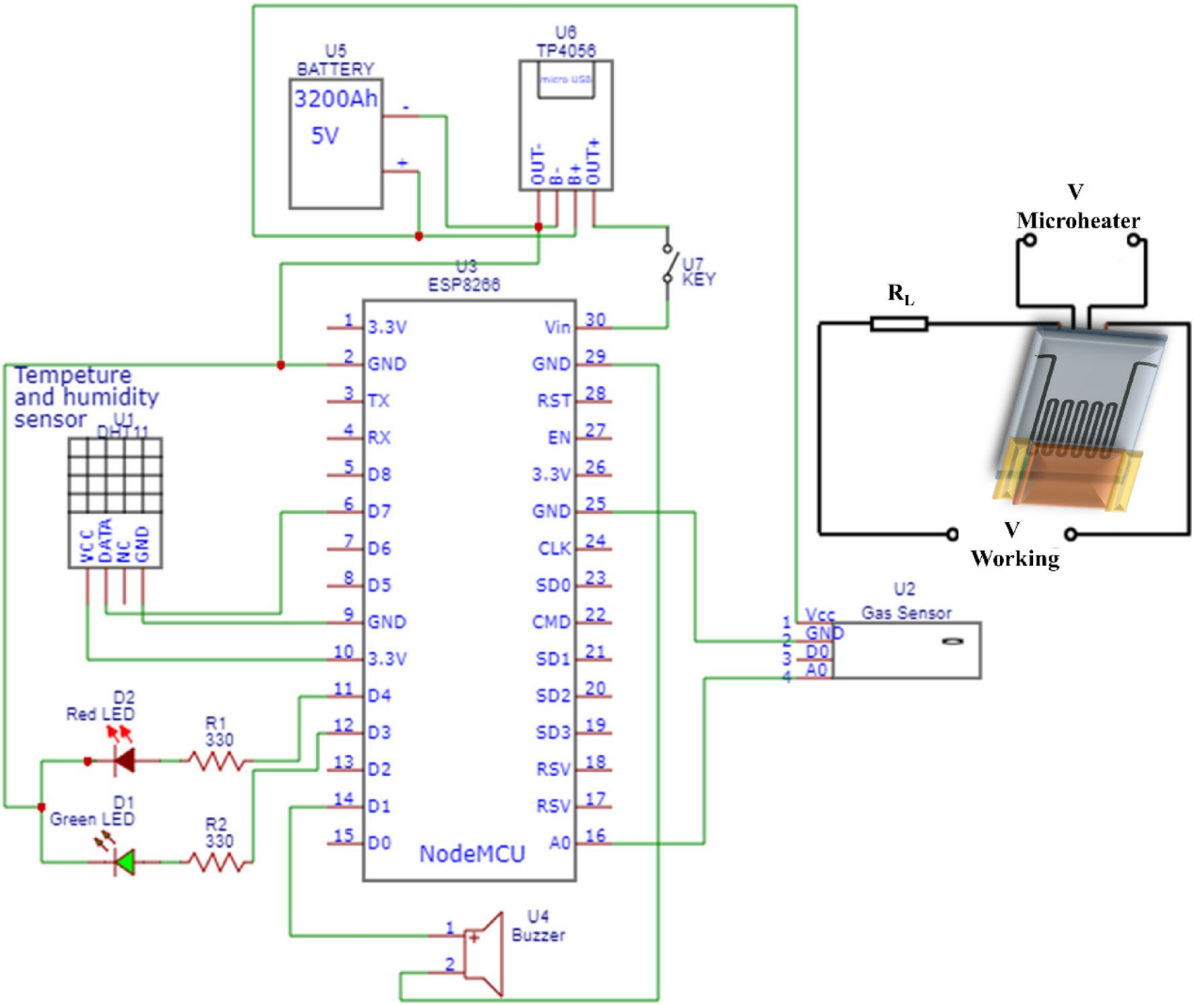
Schematic diagram depicting the circuitry of the portable device and gas sensing measuring system.

The battery supplies power to the charging module, which in turn provides power to the node-MCU ESP8266 via a switch key. The charging module also serves as a battery charger. The gas sensor is directly connected to the battery to minimize the pre-heating time. The output signal of the gas sensor is fed to the analog input pin A0 of the node-MCU ESP8266. The DHT11 sensor is powered by the 3.3 V pin of the node-MCU ESP8266 and sends its data to the digital pin D7. A green LED and a red LED are connected to the digital pins D3 and D4 of the node- MCU ESP8266 through two 330 Ω resistors. The LEDs indicate the status of the gas sensor. The alarm buzzer is connected to the digital pin D1 of the node-MCU ESP8266. All the ground pins of the components are connected to the ground pin of the node-MCU ESP8266. However, should the designed sensor detect a concerning rise in CO levels beyond 400 ppm, indicative of a potential health hazard, the system responds with a proactive alarm system. A red LED begins blinking to visually alert occupants, and simultaneously, a buzzer emits an audible warning. This dual-alert mechanism not only provides immediate notification but also caters to users with different sensory preferences. By leveraging the capabilities of the ESP8266, this integrated system not only monitors air quality but acts decisively to safeguard occupants against elevated CO levels, enhancing overall safety in the monitored space. Figure 8, displays the electrical circuit of the portable device and the designed sensor.

The web_socket_channel package, namely version 2.4.0, was used in the application development process to enable seamless communication with an ESP8266 microcontroller linked via Wi-Fi. The Android program was created using Flutter version 3.13.1 and Dart version 3.1.0. This program is essential for facilitating instantaneous data sharing by establishing a connection to a fixed IP address with a specific port configuration. The web_socket_channel primarily functions in conjunction with the stream channel class, which is an abstract class that facilitates bidirectional communication, specifically designed for web_socket_channel. Every occurrence of a stream channel offers a Stream that receives data. Conceptualize a Stream as a channel: provide an input at one end, and if there is a recipient at the other end, that recipient will receive the input. In addition to the web_socket_channel package, we utilize other commonly used packages in Flutter to develop an application that enables users to receive notifications regarding CO warnings. The code is designed to utilize a threshold to verify the level of CO gas detected by the sensor. In addition to presenting the value to the user, the code also declares a state of danger if the gas concentration exceeds a certain level. The program provides a real-time display of humidity and temperature values to the user. The URL to download and install the crated Android software.

## Conclusion

This article presents the fabrication of a binder-free P–N heterojunction gas sensor consisting of CuO/CF nanowire and hierarchical In_2_O_3_. The purpose of this sensor is to detect CO and enhance the sensor’s ability to selectively detect this gas. The findings indicate that at a temperature of 200 °C and a relative humidity of 30% at 25 °C. The hierarchical In_2_O_3_@CuO/CF sensor demonstrates the highest performance, with a response value of 10–900 ppm CO. This represents a significant enhancement compared to the pure sample. The performance of CO is greatly improved by the presence of the CuO/CF nanowire, mostly because of the greater amount of oxygen vacancies and the stronger adsorption energy on the surface of the CuO/CF layer. For portability purposes, the device is designed to be compact, and it allows each user to operate easily. This portable sensor establishes wireless connectivity with an Android application-enabled smart device.

## Experimental section

### Chemicals and apparatus

Ammonium persulfate ((NH_4_)_2_S_2_O_8_), Urea (CO(NH_2_)_2_, Hydrochloric acid (HCl), Sodium hydroxide (NaOH), Acetone (CH_3_)_2_CO, Ethanol (CH_3_CH_2_OH), Indium (III) chloride tetrahydrate (InCl_3_ 0.4H_2_O), and sodium dodecyl sulfonate (SDS) were purchased from Merck Company (Darmstadt, Germany). A CF was purchased from Nano-bazaar Company (Tehran, Iran). CO, N_2,_ and O_2_ gas (99% purity) were purchased from Saman Arak Gas Company (Arak, Iran). All other chemicals and reagents utilized were of analytical grade. Deionized water was utilized in all experiments.

Chemiresistive studies were carried out by using a digital multimeter (Victor, 88C model), and commercial power supply (TPS-3010U model). XRD diffractometer (GNR, APD 2000 PRO, Italy) with Cu Kα radiation was used to collect powder X-ray diffraction (XRD) patterns. Field emission scanning electron microscopy (FESEM) images to examine the surface morphologies and elemental analysis were recorded at 30 keV by using a TESCAN MIRA3 LMU instrument (Czech Republic). The ESP8266 microcontroller was powered by a 5-V lithium rechargeable battery (3200 A h) assembled into a battery module (T6845-C model). The portable sensor platform’s enclosure was manufactured using 3D printers, namely the L Pro model from Iran. The laser equipment utilized for the design and definition of the electrodes and heaters platform was the Rotec model from Iran, equipped with an 80 W power tube and a working table with dimensions of 60 × 40 cm.

## Materials synthesis

### Preparation of hierarchical In_2_O_3_@CuO/CF

*Step 1* The synthesis of the CuO/CF was carried out using the method described in the previous literature^55^. The CF (3 × 1 × 0.1 (thickness) cm^3^) underwent ultrasonic cleaning using a hydrochloric acid solution, acetone, ethanol, and deionized water for 10 min each, individually, in order to eliminate the surface oxide layer. Subsequently, the CF underwent a drying process in a vacuum oven at a temperature of 50 °C for 6 h. The CF that had been treated beforehand was submerged in a solution consisting of 20.0 mL of NaOH (1.25 M) and (NH_4_)_2_S_2_O_8_ (0.0625 M) for a duration of 20 min at room temperature. Following the reaction, the blue CF was extracted and rinsed with deionized water on three separate occasions.

*Step 2* The Cu (OH)_2_/CF sample was subjected to calcination in a stainless-steel autoclave at a temperature of 200 °C for 2 h. The ramp rate of the furnace was set at 5 °C min^−1^ under a nitrogen atmosphere. This process aimed to convert Cu (OH)_2_ into CuO through dehydration. Subsequently, the dark brown CuO/CF nanowires were obtained.

*Step 3* Hierarchical In_2_O_3_ was deposited onto CuO/CF using a hydrothermal method and annealing treatment. The solution was prepared by dissolving 5.0 mmol of InCl_3_ 0.4H_2_O and 8.0 mmol of SDS in 40 mL of deionized water while continuously stirring. Then, an amount of 10.0 mmol of urea was added to the mixed solution. After 1 h stirring, the initial solution was put into the stainless-steel autoclave with a capacity of 100 mL for a thermal reaction at a temperature of 120 °C. The CuO/CF that had been fabricated beforehand was submerged in the stainless-steel autoclave. The hydrothermal reaction was allowed to proceed for 12 h. The hierarchical In_2_O_3_/CuO/CF was washed with DI water and ethanol. The hierarchical In_2_O_3_/CuO/CF was dried at a temperature of 60 °C overnight and thereafter underwent an annealing procedure at a temperature of 500 °C for a duration of 5 h, with a heating rate of 1 °C per minute.

### Synthesis of pure In_2_O_3_ nanospheres

The pure In_2_O_3_ nanospheres were synthesized using the method outlined in the prior literature^36^. A solution of 5 mmol of InCl_3_·4H_2_O was initially dissolved in 50 ml of deionized water. Subsequently, 15 mmol of citric acid was added to the solution while vigorously stirring. Afterward, 30 mmol of urea was introduced into the previously mentioned mixed solution while stirring vigorously. The solution was introduced into a stainless-steel autoclave and kept at a constant temperature of 130 °C for a period of 12 h. The precipitates were collected using centrifugation, rinsed multiple times with deionized water and ethanol, respectively, and then dried at a temperature of 60 °C for a duration of 12 h. Ultimately, the In_2_O_3_ nanospheres were synthesized through the process of annealing the precipitates at a temperature of 500 °C for 3 h.

### Test set up

**Figure 9:**
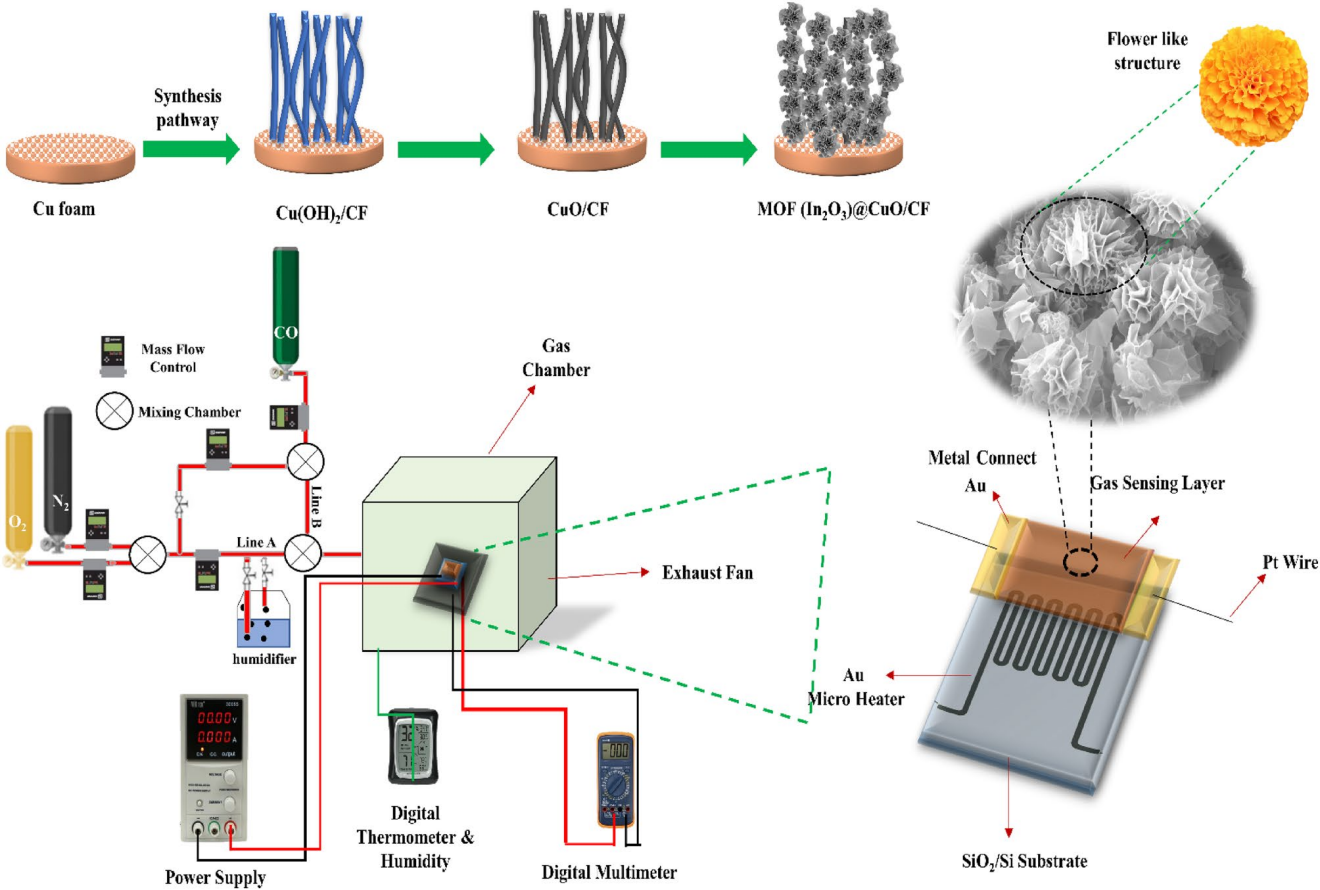
Schematic illustrating the configuration and components of the gas detection apparatus and the synthesis pathway.

As can be seen in Fig. 9, the gas detection system consists of a testing chamber, gas cylinders, mass flow controllers, a power supply, a resistance measurement device, and a digital thermometer & humidity. During the experiment, the sensor electrode and sensor gadget were placed inside a hermetically sealed testing chamber, maintaining a temperature equivalent to the ambient room temperature. Gases of N_2_, O_2_, and CO with a purity of 99.999% were added to the chamber. The RDWorks V8.0 program designed the cube-shaped part of the test chamber. The chamber was constructed by cutting plexiglass parts with a diameter of 1 cm using a laser machine. Subsequently, the chamber is assembled and the parts are sealed with adhesive. The exhaust fan and NPT Fittings, as well as the wire entrances, are entirely sealed to prevent gas leakage. The exhaust pipe is used to direct the target gas. A Glovebox-like environment has been created by employing sealed gloves, enabling us to reach the inside components of the box to turn on and off the module during testing. The chamber has a total volume of 20,000 cm^3^. The injection rates of line A and line B were independently regulated by mass flow controllers, which ensured exceptional stability and repeatability. Line A and line B are nitrogen–oxygen mixtures containing 80% N_2_ and 20% O_2_, with 1000 ppm CO added to line B. To provide various concentrations of carbon monoxide in dry and humid air:

9 $${\text{CO concentration }} = \left( {{\raise0.7ex\hbox{${V_{line A} }$} \!\mathord{\left/ {\vphantom {{V_{line A} } {V_{line A} + V_{line B} }}}\right.\kern-0pt} \!\lower0.7ex\hbox{${V_{line A} + V_{line B} }$}}} \right) \times 1000{ }\left( {{\text{ppm}}} \right){ }$$

V_line A_ and V_line B_ represent the volumes of mixture gas (20% O_2_ + 80% N_2_) and mixture gas (20% O_2_ + 80% N_2_ + 1000 ppm of CO), respectively. The calibrated gas combination of 20% O_2_ with 80% N_2_ (line A) was humidified by passing it through a manual regulator of gas flows (MRGF) and a custom-designed humidifier. In addition, CO gas was developed in four humidity conditions (40%, 50%, 60%, and 70% relative humidity at a temperature of 25 °C) by infusing a certain concentration of CO gas into a test chamber with a humid atmosphere and conducting the test after a 15 min balance. The gas flow humidity in the test chamber can be detected by sensors across the entire range of 0 to 100% relative humidity ($\pm 0.4 \% error$). Investigations were conducted using sensors under conditions of thermal stabilization, where the micro-heater resistance Rh and electric power Wh remained constant. The gas sensor materials were heated manually to a specific temperature (T). Using a digital thermometer, the temperature was regulated between 100 and 250 °C while the sensor was attached to a microheater. Every 5 s, the resistance of the sensor was recorded using a digital multimeter (DMM). Mixture gas (80% N_2_ + 20% O_2_) was injected at a steady flow rate of 1000 sccm as the background gas, establishing a stable sensor resistance baseline. The flow was then switched to the target gas with a specific concentration and mixture of gas for the gas sensing test. During the recovery stage, the gas flow was switched back to the background gas, and the resistances of the sensors were restored. The sensor’s response to different gases was examined to confirm its selectivity. Following the sensor’s resistance stabilized, a certain volume of testing liquid was introduced into the 20 L gas chamber using a syringe. The liquid was heated and evaporated to produce a precise quantity of test gas in the form of volatile molecules. The gas concentration of Volatile Organic Compounds (VOCs) in the chamber was calculated using Eq. (10).

10 $$C = 22.4 \times d \times \rho \times V_{1} \times 1000/\left( {M \times V_{2} } \right)$$

The desired gas concentration (C) in (ppm) is calculated using the liquid purity (d) in percentage, liquid density (ρ) of the gas in (g/mL), liquid volume (V_1_) of the target gas in μL, molecular weight (M) of the target gas in (g/mol), and chamber volume (V_2_) in liters.

## Data Availability

The datasets supporting the conclusions of this article are included within the article.
